# Designing internet-enabled patient education for self-management of T2D diabetes—The case of the Razavi-Khorasan province in Iran

**DOI:** 10.1371/journal.pone.0250781

**Published:** 2021-04-27

**Authors:** Javad Jafari, Klas Karlgren, Hossein Karimi Moonaghi, Parvin Layegh, Stefano Bonacina, Italo Masiello

**Affiliations:** 1 Department of Learning, Informatics, Management and Ethics, Karolinska Institutet, Stockholm, Sweden; 2 Education Development Center, Mashhad University of Medical Sciences, Mashhad, Iran; 3 Department of Research, Education, Development and Innovation, Södersjukhuset, Stockholm, Sweden; 4 Faculty of Health and Social Sciences, Western Norway University of Applied Sciences, Bergen, Norway; 5 Department of Medical Surgical Nursing, School of Nursing and Midwifery, Mashhad University of Medical Sciences, Mashhad, Iran; 6 Endocrine Research Center, Imam Reza Hospital, School of Medicine Mashhad University of Medical Sciences, Mashhad, Iran; 7 Faculty of Technology, Department of Computer Science and Media Technology, Linnaeus University, Växjö, Sweden; Chiang Mai University Faculty of Medicine, THAILAND

## Abstract

**Background:**

The number of people with diabetes is estimated to increase to 642 million by 2040, with most having type 2 diabetes. Patients with diabetes require continuous monitoring and possible treatment changes. Patient education is the process of enabling individuals to make informed decisions about their personal health-related behaviours and internet-enabled interventions have the potential to provide support and information to patients with diabetes.

**Objective:**

The aim of the study was to design a portal prototype based onto two models of care and a contextualised education programme to support the self-management of diabetes patients by involving stakeholders in the Iranian province of Razavi-Khorasan.

**Methods:**

A Design-Based Research framework was adopted. A qualitative research method was used to analyse interviews with patients and care givers. Mock-ups were developed first and designed with features of user-driven and self-care models of care. The mock-ups also had adaptation features, such as for control of the disease, ability to cure self, and family support. The portal prototype was developed iteratively by building on the mock-ups and evaluated through interviews. The features and elements of the mock-ups and the portal prototype were evaluated in an outpatient diabetes clinic in Mashhad.

**Results:**

Thirty-three participants were involved in the study. The evaluation of the mock-ups resulted in two themes and seven categories: 1) self-care improvement, including self-care requirements and self-management, and 2) educational usefulness, including medical information, information mode, mobility, interaction, and efficiency. The mock-up evaluation was used as a basis for designing a portal prototype. Next, the portal prototype was evaluated, and three categories emerged from the interview data: 1) user experience, 2) functionality, and 3) interactivity. Participants were not able to prioritise between the two care models. Some functionalities of the portal could benefit from the development within a cultural context to determine differences to the best way to present material.

**Conclusions:**

A portal prototype has been designed to include two care models to support self-management and functionalities that support aspects of culture-specific diabetes self-care. This study provides guidance on developing an internet-enabled educational portal, aimed at providing support for patients in their social context.

## Introduction

The number of people in the world with diabetes today exceeds 422 million and is estimated to increase to 642 million by 2040 [1]. About 90% of all diabetes cases are of Type 2 (T2D), about 80% of all patients with diabetes live in low- and middle-income countries, and the large majority are in middle adulthood [2, 3]. The prevalence of diabetes in 2011 in Iran was reported to be 11.9% in adults aged 25–70 years [4] and 14%-16% in the age 50–60 in the Razavi-Khorasan province [5]. Unhealthy diets, obesity, and sedentary lifestyles are three major factors that contribute to the diabetes epidemic [6]. A study showed that just over a decade ago the prevalence of overweight or obesity in the Razavi-Khorasan province was 40.6% in elderly people [7]. Unfortunately, there is no cure for diabetes, but it can be delayed and its complications prevented by changes in lifestyle measures such as a healthy diet, moderate physical activity, weight control, and cessation of tobacco use [1]. Therefore, many of the interventions for the prevention of diabetes complications target the education and information provided to patients with diabetes to aid the self-care process of the disease.

Terms like “self-care” and “self-management” are extensively used in the growing scientific literature about diabetes patient care. In self-care, the focus is on prevention of disease or maintenance of a well-being state, while in self-management, also self-care management, focus is on coping with the disease or controlling parameters [8]. These terms are also used throughout this paper.

## Patient education

Patient education is important for empowering patients with diabetes to effectively take care of their health. There are many tools and resources for diabetes self-care and self-management available on the internet. Those are made by hospitals, universities, or patient organisations. A recognised education programme is the Diabetes Self-Management Education (DSME). DSME has elements of care for patients with diabetes and helps educators and care givers in delivering evidence-based education and self-management support [9]. DSME follows theories and standards to help patients improve their self-management and to enhance their clinical outcomes and quality of life by helping them change their behaviour, that is, to stop smoking, lower their alcohol consumption, improve their diets, increase physical activity, and intensify self-monitoring [10]. In fact, successful DSME can provide patients with the knowledge and skills necessary to delay prolonged diabetes-related complications [9, 11, 12]. However, educational programmes often do not address cultural needs, which may influence health behaviour in individuals in a specific cultural context [13]. For those reasons, a comprehensive programme called Persian Diabetes Self-Management Education (PDSME), a culturally sensitive version of the DSME curriculum, was designed for patients with diabetes in Iran [13]. Studies show that characteristics of patients are culturally specific and can influence diabetes education [14–16]. Shekibazadeh et al. [17] determined that besides common aspects of living with diabetes, there are also a number of cultural-specific diabetes self-care aspects: 1) prioritising the family before self; 2) experience of stigmatisation; 3) strong social relationship and group pressure; 4) spiritual beliefs that relate to health beliefs; 5) wide application of alternative and herbal medicine; and 6) negative perceptions of Iranian medicine. Based on a number of behaviour-change theories, PDSME has been proven to bring improvements in Glycated haemoglobin (HbA1c) levels, self-care behaviours, and knowledge about diabetes in a population of 350 newly-diagnosed T2D patients [13].

## Contribution of technology to self-management

Internet-enabled interventions, either on computers, tablets or smartphones, are now additional and even alternative providers of education and support for patients with T2D. They show favourable outcomes and also significant improvement of self-management and glycaemic control of T2D [18, 19]. Information technology-based interventions can also improve well-being outcomes [20]. Several successful educational programmes have focused on empowering patients with diabetes through technology support [21], and two care models have been widely used. Specifically, the user-driven model of care management involves patients’ and health care professionals’ joint participation in decision-making across electronic media [22]. Collaboration about various aspects related to a chronic disease and the definition of a set of digital interventions comprise this model. The other approach is for patients to access the relevant knowledge, preferences, and values through technology that can enable them to make decisions on their own and carry out specific actions–the self-care model [23]. The self-care model also involves following recommendations of healthcare providers about treatment and lifestyle to enhance self-management [24]. There are differences between the two models. Decision-making is emphasised as being shared in the user-driven model, while communication technology and discipline–defined as the human capability to carry out specific tasks–help patients attain better health outcomes and are built into the self-care model. Regardless of the model, the use of internet-enabled devices by patients with diabetes can be a key step forward for diabetes self-management by possibly improving the quality of life [25]. Patient portals are a widely adopted internet-based solution for patients’ access to their health information [26]. A recent review has shown that there is variability in features displayed across the portals and diabetes outcomes, which makes it difficult to draw conclusions about the effectiveness of the portals in achieving changes to diabetes management [27]. Although the majority of T2D patients in Iran still prefer more traditional methods such as radio and television to obtain information, the use of technology and the incidence of diabetes in the country have been increasing [28]. This necessitates research to understand the changing self-care and self-management patterns [29]. But there is a lack of studies in the context of Iran on how to design educational and supportive technology-based solutions, also involving healthcare providers, to help the patients gain better control of the disease.

### Design-based research approach

Educational practitioners have often complained that there is a gap between educational research and educational practice; the Design-Based Research (DBR) methodology can help bridge that gap [30]. The five characteristics for DBR are: 1) It takes place in continuous cycles of design, evaluation, and redesign; 2) It takes place in real-life settings, not only in laboratories; 3) It is aimed both at testing and refining theoretical principles and also advancing practice; 4) It is characterised by mixed-methods (not just a single method) of inquiring; and 5) Designers, researchers, and practitioners with different expertise work closely together in designing, evaluating, and redesigning [31]. The objective of DBR is cooperation in the design of ‘user-centred’ information systems and services in real settings [32]. Also, previous studies have shown that involving patients as co-designers may be useful in providing content which is experienced as meaningful in educational websites [33]. It is not well-known how the two care models, i.e., user-driven model, and self-care model, could be implemented in a portal for diabetes care in a culturally specific context, such as Iran. If new technologies are not tailored to the needs of the diabetes patients, they risk being left unused, and ultimately some patient groups may not receive the support they need, jeopardising their health. To our knowledge, DBR has not been yet applied to the development of a patient portal by involving patients, doctors, and nurses in Iran.

The overall aim of the study was to increase the understanding of how to design a portal prototype to support the self-management of diabetes patients in a specific cultural context in Iran (the case of the Razavi-Khorasan province).

## Methods

### Study design

This is a formative research study using a DBR process. The objectives of the study were to:

- Use a Design-Based Research process to test, evaluate, and update the mock-ups and portal prototype through users’ feedback and reactions.
- Design an interactive portal relevant in Iran and for its T2D patients incorporating both models of care (user-driven and self-care models)

The researchers used qualitative research methods–semi-structured interviews and observations of participant using the prototype. Following the iterative process of DBR, we developed a series of mock-up and then collected the participants’ feedback and reactions to the mock-ups first and to the portal prototype later.

### Sample and clinical setting

The researchers recruited three types of participants using a convenience-sampling approach, for a total number of 25, divided in the two phases (Table 1):

**Table 1:** The actual numbers of participants in the study.

Participants	Phase 1. Mock-ups	Phase 2. Prototype
T2D patients coming to the clinic in Mashhad	6 patients (3 women, 3 men)	19 patients (9 women, 10 men) (4 of which were the same as in Phase 1)
Clinic nurses working as nurse educators	2 nurses (2 women)	2 nurses (same as in Phase 1)
Physicians treating the patients	2 physicians (2 men)	2 physicians (same as in Phase 1)

Inclusion criteria for patients were: confirmed diagnosis of T2D at least one year prior to the study, having access to the internet at home or at the workplace, having access to a smart phone, having more than nine years of formal education, and being cognitively and physically able to participate personally in the study. The one inclusion criterion for the physicians and nurses was having more than five years of experience working with patients with diabetes.

The outpatient clinic, the specific context where the research was conducted, had about 8,000 patients visiting regularly. The clinic was private but under the control of a medical university and located in Mashhad, a large Iranian city with about three million inhabitants that is located in the Razavi-Khorasan province with a population of around six million people. All interviews were conducted in a separate room of the clinic and at a convenient time for the participants. The interviewers were the first author in Phases 1–2, who had worked in the diabetes clinic for about six years, and, only in Phase 2, the software engineer who developed the portal.

The consolidated criteria for reporting qualitative research (COREQ) were used in this paper. This is a formal reporting checklist built on common methods used for data collection in qualitative health research [34].

### Phase 1. Mock-ups

#### Design of the mock-ups

The mock-ups were designed and developed by a computer engineer, himself a diabetes patient, who followed the characteristics of the two care models (Table 2) and the DSME programme [35]. The first author also contributed to the design of the mock-ups and the PDSME programme with his experience as a medical doctor working with patients with diabetes in the specific Mashhad clinic and his previous research [28, 29]. The results from the previous research showed that the participants’ prioritisation of DSME topics were too different to possibly identify only a single ideal model to use in the design of the mock-ups, therefore both models were incorporated in the mock-ups [35]. Examples of DSME topics that were more strongly related to the models are shown in Table 2.

**Table 2:** The main characteristics of the two care models.

Model	Main Component	Strategies	Outcomes	DSME topic
User-driven model [22]	Shared decision-making	Collaborative learning between multiple users: stakeholders, patients, health professionals	Information technologies increase connectivity between patients and health professionals	Disease and treatment process; Developing strategies to address psychosocial issues
Self-care model [23]	Communication technology	Problem detection, functional relationships, and problem-solving	Continuous reflection, problem detection, active engagement, and increased control of the chronic condition	Monitoring blood glucose and interpreting the results; Prevention, detection and treatment of acute complications

A total of 22 mock-ups plates were designed during six work meetings between the first author and the computer engineer. The mock-up plates had buttons, links, text, and pictures just as in a regular internet portal but without the interactivity, and they were shown to the participants on a laptop computer. By following the educational guidelines of the PDSME programme, we made sure also to feature tools that could be used for the ability to control the disease, ability to cure self, and family support to incorporate some of the culture-specific self-care aspects, as explained by Shekibazadeh et al. [13]. Therefore, the mock-ups included tools, such as chat room with care givers, family members, and fellow patients; self-care tools, such as a knowledge assessment test, a “help me” feature, progress reports based on laboratory results, self-care videos, and links to other source of self-care instruments; and educational tools, such as a curriculum and educational programmes, videos about body organs, laboratory analysis and more. The above tools are not different from tools of other existing diabetes portals [36], however the use and usefulness of the tools in the social context are related to the culture of the patient. The mock-up plates illustrated also personal information and covered aspects related to diabetes management to demonstrate how the portal would work.

#### Evaluation of the mock-ups

The first author conducted semi-structured, face-to-face individual interviews to collect users’ attitudes and elicit feedback on the mock-ups, with the scope of understanding what could have been done that might have engaged them in using a presumptive portal. A protocol was followed to investigate various subjects [34]. The interviews were conducted in Persian by using the protocol that had been pilot tested with two diabetes patients. All interviews were digitally recorded and the audio file transcribed verbatim, and each interview lasted between 30–45 minutes. A quick analysis of the interview data was conducted by two of the authors (JJ, HKM) right after each interview. The authors decided that saturation [37] had been reached when additional major design and usability issues—especially those pertaining to self-management—were no longer brought up by the users; with six patients, two nurses, and two physicians (10 participants). Codes were defined and subsequently translated into English. Codes were derived from the text data. During the inductive content analysis, the data were analysed thoroughly and sub-categories, categories and themes were generated [38, 39]. Consensus was reached through discussions with the co-authors to confirm the consistency of the findings. Data for this phase were collected in August 2016.

Each interview was considered to be a “microcycle” of the design process [40]. This process of cycles was meant to help improve the artifacts in the portal prototype.

### Phase 2. Portal prototype

A software engineer designed the portal prototype based on the evaluation of the mock-ups. The engineer then conducted another round of semi-structured, face-to face individual interviews with 19 patients, two physicians, and two nurses. The interviewer used an interview guide that was pilot-tested with two diabetes patients. The feedback from the testing led to rephrasing some questions before starting the evaluation of the prototype. The interview data of the evaluation were collected as audio files and transcribed verbatim. The questions focused on the user experience of the design, patients’ needs, interactivity, accessibility, and privacy. The data were analysed, coded, and categorised independently by two authors (JJ, HKM) using inductive content analysis [41]. Consensus was reached through discussions between the co-authors to confirm the consistency of the findings. Data for the third phase of the study were collected from October to November 2017.

### Ethical considerations

All participants in the study received oral and written information about the study aim and read and signed a consent form. Participants were assured about their anonymity and the confidentiality of the collected data. They were also informed of their right to withdraw from the study at any time. The study was approved by the Ethical Committee of Mashhad University of Medical Sciences (IR.MUMS.REC.1395.108).

## Results

### Phase 1. Mock-ups

The first author interviewed 10 participants (five men and five women, with an average age of 43 years, ranging from 27 to 57 years): six patients, two nurses, and two physicians. One patient did not want to sign the consent form and was excluded from the study. The researchers derived 326 codes, which resulted in 14 sub-categories, seven categories, and two themes–self-care improvement and educational usefulness, as shown in Table 3.

**Table 3:** Sub-categories, categories, and themes derived from the interview data in Phase 1.

Sub Categories	Categories	Themes
Nutrition	Self-care requirements	Self-care improvement
Physical activity		
Complications	Self-management	
Stress		
Educational content	Medical information	Educational usefulness
Accuracy		
Educational film	Information mode	
Features		
Customisation		
Mobile and applications	Mobility	
Patient engagement	Interaction	
Family and peer support		
User interface	Efficiency	
Access to internet		

#### Self-care improvement

Self-care improvement was the theme to which participants contributed the most feedback. This theme included two categories: *self-care requirements* and *self-management*. *Self-care requirements* referred to nutrition and physical activity, which are the most essential self-care behaviours for patients with diabetes to prevent complications. All participants expressed their interest in what was shown by the mock-up plates, and patients wanted to know more about aspects related to diabetes, emphasising the need for these subjects to be included in the portal.

*“It is good to know how much walking I have to do per day*, *how much exercise I need to do”*. (Patient 4, female, 51 years old)

The categories brought up the importance of learning more about nutrition and exercise. From the mock-ups, patients became interested in knowing about calorie intake, dietary supplements, eating fruits and vegetables and the benefits of physical activity. Physicians and nurses mentioned these as important for patients. One of the physicians stated that patients could decrease diabetes complications by changing their behaviours.

*“Changes in diet and physical activity increase the ability to deal with the complications of diabetes*. *This information is important for the patients to know”*. (Physician 1, male, 47 years old)

The *self-management* category referred to the process of assisting the individual in taking responsibility for their own health–for example, by knowing more about complications and stress. This demonstrated the importance of these features in a mock-up.

*“Mentally*, *one must be comfortable*, *and it is very important that a patient can control himself and solve problems*, *and it is important to manage our stress”*. (Patient 2, female, 47 years old)

After looking at the mock-ups, a nurse also mentioned that the management of nutrition and stress were important for patients.

*“In my opinion*, *both nutrition and stress are very important in diabetes*, *and [patients] should work on them”*. (Nurse 2, female, 28 years old)

#### Educational usefulness

This theme consisted of the following categories: *medical information*, *information mode*, *mobility*, *interaction*, *and efficiency*. The *medical information* category showed participants’ desire to receive accurate educational content related to the disease, as exemplified by the mock-ups.

*“I like information about drugs*. *I also want to know how I can decrease my cholesterol level”*. (Patient 7, female, 52 years old)

The *information mode* category was related to how information should be presented and included, such as in an educational film, as well as its features and customisation. These represented aspects of how users would like to get information related to their chronic disease. Upon looking at the section with educational video clips, a physician reflected that these are attractive for patients and that patients may like the ‘question and answer’ feature represented in the mock-ups.

*“The most important and welcome things for patients are the question and answer sessions with the educational videos”*. (Physician 2, male, 50 years old)

Participants talked about their experience with using other websites and the internet to communicate with other patients and find information about diabetes. Some patients showed strong feelings about the portal portrayed by the mock-ups because they could find information related to their personal situation through the customisation feature.

*“That’s what I was looking for*! *For them [the patient pointed at the Information feature]*, *I did not know about this information about diabetes and now I can see all of them in one place”*. (Patient 3, male, 38 years old)

In the *mobility* category, the mock-ups inspired some patients to say that they were happy to use mobile phones to get information related to diabetes. They stated that a mobile application would have been a better portal than a computer web portal since it can be reached easily and at any time, especially when it is really needed.

*“My phone is always with me*. *Now I can buy the internet [surf time] and always be connected through my mobile”*. (Patient 6, male, 22 years old)

The *interaction* category consisted of patient engagement as well as family and peer support. In patient engagement, participants emphasised different ways to engage the patients to use the portal in the future.

In the family and peer support sub-categories, patients insisted on family and peers having a role in the portal to help them gain better control of their disease.

*“Children are using the internet more these days*, *and they can help us use the portal*. *I am helping my other family member who has diabetes*, *and through this portal*, *I can help a lot of people and peers and share my experiences”*. (Patient 4, female, 51 years old)

The *efficiency* category consisted of two sub-categories: user interface and access to the internet. Some participants recommended making some changes to make the prospective portal more efficient and less generic, such as changing the photos that the researcher used as symbols and adding more links and other information to make it is easier to understand and more contextualised.

The patients thought that the portal portrayed by the mock-ups would be interesting and not complicated to use; therefore, they were interested in using it in the future.

*“It is simple and does not involve too much work to use*. *It is no problem*. *There are good informational texts*, *and they are readable [understandable]”*. (Patient 1, female, 43 years old)

One physician and one nurse complained about the problems that patients have these days using the internet in Iran. They mentioned that, although the internet is not so popular yet, access to the internet will improve in the future.

*“Large problems for our patients are access to the internet*, *a slow connection*, *and that the internet is not always available and has too many connection drops”*. (Physician 1, male, 47 years old)

The mock-ups were simple and did not include all educational subjects and aspects important for patients. In Phase 1, it was not possible to discern which of the two models was preferred by the participants. Four categories, i.e., *Self-care requirements*, *Self-management*, *Information mode*, *and Interaction*, related to culture-specific aspects of diabetes self-care, as they considered several patients’ behaviours in the social context, such as self-care behaviours, health information gathering, and engagement with family and peers.

### Phase 2: Portal prototype

A portal prototype was designed by two computer programmers and the first author. Based on the previous iteration, all Self-care improvements and a number of Educational usefulness features were designed into the portal prototype. That is, all topics of the DSME and one topic of the PDSME were included. Those were: Disease and treatment process; Nutrition management into lifestyle; Physical activity into lifestyle; Using medication safely; Monitoring blood glucose and interpreting the results; Prevention, detection, and treatment of acute complications; Prevention, detection, and treatment of chronic complications; Developing strategies to address psychosocial issues; Developing strategies to promote health and change behaviour; and Application of alternative and herbal medicine. We also added educational subjects, made interface changes, and made the portal less generic. In addition, we designed it to fulfil the characteristics of the two models from the start, as the results of the first iteration did not show that patient preferred one model over the other. Therefore, the prototype contained eight educational courses on a number of topics, including the effect of high blood glucose on body organs and herbal medicine. Results of laboratory tests could be uploaded to the system at any time, and lab result outcomes calculated and displayed. An online chat feature was available for use between patients and nurses and/or physicians. Computational tools were added, such as a food calorie counter and a calculator for checking body mass index and glycaemic index for different types of food. A self-educational feature was also present—‘Help me’.

Twenty-three participants (14 men and 9 women: 19 patients, two nurses, and two physicians) were interviewed to assess the portal prototype and asked about what could be done that might engage them more in the use of the portal. Also in the phase, one patient did not want to sign the consent form and was excluded from study. The interviews resulted in 150 codes, 12 sub-categories, three categories, and no particular theme. The categories and sub-categories are shown in Table 4.

**Table 4:** Sub-categories and categories derived from the interview data in Phase 2.

Sub-categories	Categories
Satisfaction	User Experience
Willingness to use	
Attractive	
Up-to-date information	
Pedagogical information	Functionality
Guidance	
Simple access	
Medical information	
Visual presentation	
Communication	Interactivity
Feedback	
Reminder/Alerts	

#### User experience

The *user experience* category included four sub-categories: Satisfaction, Willingness to use, Attractiveness, and Up-to-date information. Participants showed their satisfaction with the portal in its current state and emphasised the importance of having up-to-date information. Many participants mentioned that the portal was ready for use and that they wanted to use it as soon as possible. Other participants suggested how to make the portal more attractive, and some participants asked how they could find it on the internet.

*“I think it was perfect and [contained] good information on diabetes*. *It was complete compared with the one before* [compared to the mock-ups] *… what I saw before”*. (Patient 17, female, 25 years old)

*“Overall*, *the portal is good and details can be added over time”*. (Physicians 1, male, 47 years old)

Some participants said that the portal should be kept up to date by making it available, and possibly it should contain active contact information of the healthcare givers, while responses to the online questions should be prompt.

*“The site should be updated frequently and a physician or nurse should be online”*. (Patient 11, male, 37 years old)

#### Functionality

This category included five sub-categories: Pedagogical information, Guidance, Simple access, Medical information, and Visual presentation. Within the *functionality* category, participants insisted on the importance of providing pedagogical information in the portal. They requested more medical information to be added and asked for guidance tools. They also expressed the need to get easy access to the portal and obtain medical information.

They provided suggestions on how to make some visual and structural modifications. The participants pointed out that the terms presented in the portal should be uncomplicated and pedagogical and that the whole portal should be made as simple as possible to use. They also recommended adding explanations to parts of the portal and adding information about the laboratory history.

*“The educational material should not be hard to understand*. *Try to make it simple and pedagogical*. *It is better to make things simpler and not use medical terminology”*. (Patient 16, female, 38 years old)

Participants said that the portal provided them with medical information and instruments for managing their diabetes. One patient stated,

*“The information about organs is useful and helps us know what to do to prevent damage of the organs when suffering from diabetes”*. (Patient 6, female, 55 years old)

Patients suggested adding links, icons and clearer pictures and using diagrams for a better overall visual presentation.

*“The photo [icon] for online chatting should be like two people speaking to each other”*. (Patient 4, male, 52 years old)

#### Interactivity

The *interactivity* category consisted of three sub-categories: Communication, Feedback, and Reminders/Alerts. Participants mentioned the need to have contact with doctors and nurses. They felt that the portal would be helpful because it would allow them to ask questions and get feedback so that they could learn more about their health situation and progress. The “Help me” feature was able to help patients to understand their current situations based on their medical condition. This feature interested the patients and three of them mentioned that they learned so much from it and felt so knowledgeable so that they felt like being physicians. The patients also suggested that the portal should give them reminders/alerts to help them remember when to book visits with their physician.

*“It is very good that we can be reminded to have contact with our* physicians *and nurses and to ask questions at any time”*. (Patient 15, female, 30 years old)

Suggestions and feedback about the portal were of practical nature. For example, participants wanted to know the approximate cost of lab tests and diabetes medications since they changed often. Participants also expressed the wish to possibly book an appointment with their physician through the portal.

In this phase, two culture-specific aspects surfaced from the interviews. The first was related to the *functionality* category, where the sort of information, the access to it, the guidance around it, and the visual presentation of it is steered by a cultural context. The second was related to the cost of treatment not covered by the insurance system.

## Discussion

In this study, we used the DBR process to increase the understanding of how to design an educational portal for patients with diabetes that was based on two care models and tested in the cultural context of the Razavi-Khorasan province in Iran. The iterative phases characteristic of DBR helped the researchers move from a design to learning about the design and artifact improvements (Phases 1 and 2). Each iterative phase confirmed or updated the results from the previous iterations–that is, which information, features, and artifacts provided in the mock-ups and portal were positively acknowledged. This study also confirmed the design based on a combination of the user-driven and self-care models [35]. In addition, this study confirms a number of aspects of culture-specific diabetes self-care shown in the literature [17], as for example strong social relationships and treatment costs.

The DBR approach recommends involving users during the design of the educational artifact to strive for user-centric end-products [32]. Many of the study participants declared their satisfaction with both the mock-ups and the portal prototype. The study findings confirmed that patients taking part in co-design can facilitate the addressing of specific topics to make the design significant and meaningful for the end-user. This becomes critical when there are few educational programmes in Iran and most of them are physical, on site programmes located in Tehran [13]. The results from our study have highlighted how important it is to follow the users’ needs when designing a product meant to have practical value in a specific context and that can be an important complement to existing programmes.

In Phase 1 of this study, the participants’ comments fell within two major themes: self-care improvement and educational usefulness. The participants expressed the needs associated with diabetes, such as healthy nutrition and physical exercise, related to self-care improvements and how those needs should be met in the portal to help improve self-care. In line with our results, researchers have highlighted the importance of nutrition and physical exercise as areas of T2D to diminish the risk of complications and stress [26]. The participants also expressed satisfaction with online access to information related to diabetes, how the information is presented, its educational and personal value, and its accuracy–all of which can help patients in understanding and managing their disease. With a user-centred approach of Phase 1 –in our case involving patients, nurses, and physicians–the researchers and developers were able to later design a portal prototype that was culturally sensitive and usable by a population of patients still at the outskirt of internet-enabled self-management but ready to take the next and almost necessary step into it. In fact, just as it is the case of diabetes portal in industrialised countries, diabetes care is more readily accessible on-line and availability of patient health records in the USA is estimated at 75% by 2020 [27], while adoption is much lower in low-and middle-income countries, 15%-35% respectively [42], including Iran [43].

The user interface portrayed by the mock-ups seems to have been accepted by the participants. Useful design input was obtained, although at times contradictory. On the one hand, the participants emphasised accuracy of the information, but on the other hand, they wanted the educational material to be simple and without medical terminology, which is a tension that has been noted in previous studies [33]. There is not seldom a conflict between, on the one hand, user requirements for a simple, intuitive design making use of everyday language; and on the other hand, requirements for accurate and complete medical information [33]. This is a contradiction that designers have to deal with; it may be tempting to choose simplicity as this is often asked for, but this may in the long run not meet the needs of the users. Similarly, we had seen that the participants were unable to decisively prioritise between care models and at times proposed requirements which were not realistic such as always having a physician or nurse online to answers questions. So, while the involvement of the participants in the DBR has been valuable, designing has not been just a matter of collecting a list of requirements from the participants, but also a matter of making design decisions which have involved weighing the advantages and drawbacks of different design choices.

In Phase 2 of this study, participants’ needs and expectations helped the researchers design the prototype to be easily understandable, with limited amounts of medical jargon, and easily accessible by the target audiences with regard to form and content. Based on the authors’ previous research [29] and the results of the current study, the patients seem to need deeper knowledge and skills to understand and manage their diet. Therefore, the researchers added different tools in the prototype, one of which helped the patients count the calories of different types of food. A contextual feature that can be added to the prototype is the approximate cost of lab tests and diabetes medications. Those change often in Iran, while treatments are routine in other countries [17].

The patients also stated how both medical and pedagogical information should be presented, visualised, and communicated between the health care provider and the patient. These are all necessary aspects of the portal prototype that can enhance overall satisfaction with care, expand access to diabetes information, enhance patient-provider communication, and possibly even improve clinical outcomes [44]. A recent review claimed that patients who are interested in and actively seek access to information related to diabetes are likely to have better control of their blood glucose; therefore, internet-enabled interventions have the potential to improve clinical outcomes [45]. However, strategies may be needed for adapting information and portal functionalities to a specific cultural context.

The portal prototype emphasises specific technological and educational aspects, which combined could simplify and sustain users’ engagement. For example, the user-driven model involves patients’ collaboration with health care professionals across an IT system, while the self-care model enables them to make decisions on their own. Features of both models of care were welcomed by the participating patients in this study, as research for each separate model [22, 23] and combined models has shown [35]. Lack of family support is identified as a main barrier to self-care in Iran [13] and this is also something that was requested in our study.

### Limitations

A limitation of this study is that the participants are from one diabetes clinic and therefore not representative of all patients with diabetes in Iran. However, they share a similar culture, problems, and needs. Another limitation is that the endocrinologist was not included from the beginning of the study but was only involved after the prototype was developed. Involving her earlier might have provided useful medical input in the early design phases.

## Conclusion

There has been an increase in internet-enabled services for diabetes’ self-care. With the help of relevant stakeholders, this study shows that a portal for diabetes self-care should be designed to address a number of health-related behaviours in a specific cultural context. Participating T2D patients identified several such behaviours, such as self-care behaviours, health information gathering, and engagement with family and peers. Local practitioners should be aware that portals that address several health-related behaviours through self-care models may reach more patients and enhance the overall patients’ satisfaction with care and possibly even improve patient clinical outcomes. Based on the study results, the researchers aim to continue the development and implementation of an educational diabetes portal in Iran. A usability study of the portal will be conducted with the ultimate objective of improving diabetes outcomes.

## Supporting information

S1 File (PDF)
