# Supplementary material for: Designing internet-enabled patient education for self-management of T2D diabetes—The case of the Razavi-Khorasan province in Iran
Source: PLoS One. 2021 Apr 27;16(4):e0250781. doi: 10.1371/journal.pone.0250781 (PMC8078778; doi:10.1371/journal.pone.0250781)
Supplement: S1 File — (PDF) [file pone.0250781.s001.pdf]

سئوالات مصاحبه مرحله سوم از بیماران:

نظر شما در خصوص اطلاعات فردی چیست آیا مواردی هست که باید حذف یا اضافه شود؟

نظر شما در خصوص ورود اطلاعات نتایج آزمایشات چیست؟

نظر شما در مورد شکل ظاهری و جذابیت و نیز سهولت دسترسی به مطالب آموزشی و ابزارها و شیوهای آموزشی و ارتباطی ارایه شده چیست؟

نظر شما در خصوص پیش آزمون و پس آزمون ونیز متون آموزشی. فیلم آموزشی. محیط گفتگو با دیگر بیماران. پرسش و پاسخ چیست؟ چه شیوهای دیگری باید لحاظ شود؟

نظر شما در مورد قسمت کمکم کنید چیست؟

نظر شما در خصوص پیشرفت سلامتی و لینکهای چیست؟

نظر شما در مورد محاسبه گرهای مفید چیست؟

آیا فکر میکنید این وب سایت نیازهای آموزشی شما را براورده میکند؟

جالب ترین قسمت از نظر شما کدام قسمت سیستم بود؟ کدام قسمتها جالب و کاربردی به نظر نمیرسند؟

چه قسمت هایی از سیستم آیا شما فکر می خوب طراحی شده اند؟

چه بخش هایی از سیستم آیا شما فکر می ناقص طراحی شده بودند؟

چه اطلاعاتی شما برای کنترل بهتر بیماری نیاز دارید که در اینجا لحاظ نشده است؟

چه پیشنهادات شما برای بهبود سیستم دارید؟

All Codes (Patients, Nurses and doctors)

	categories	Sub Categories	codes	codes
improvement Self-care	Self-Care illness requirements	Suitable nutrition in diabetes	آموزش تغذیه و کالری مصرفی D124	علاقه مند به دانستن میزان قند میوه هاP24
			تشخیص دادن میزان کالری عذاهاD126	مهم بودن رژیم غذاییP27
			تاثیرداشتن تغذیه در کنترل دیابتD127	مهم بودن رژیم غذاییP29
		تغذیه صحیح در دیابت	سئوال داشتن در خصوص تغذیه کمکیN144	سئوال داشتن در مورد تغذیهP317
			اضافه شدن دیابت در سفرN152	دانستن میزان قند غذاهاP318
		جایگزینهای غذایی	اضافه شدن بشقاب مدیترانه ایN153	چه غذاهایی بخوریمP321
			سئوال داشتن بیماران در مورد تغذیهN28	علاقه داشتن به گرفتن رژیم عذاییP330
			سئوال کردن بیماران از جایگزینها غذاییN238	علاقه به دانستن میزان مصرف مواد غذاییP49
			سئوال کردن بیماران از واحدهای غذاییN239	علاقه به مقدار کالری مصرفیP414
			بیشترین سئوال بیماران از تغذیهN21	قرار دادن رژیم غذاییP67
			بیشتر در مورد تفذیه میخواهند بدانندN22	داشتن اطلاعات در باره میوه هاp112
			دانستن جایگزینهای تغذیه N23	نداشتن تغذیه مناسب به علت نوع شغلp113
			مهم بودن تغذیه در طول روز برای بیمارانN24	مهم بودن غذاP212
			مهم بودن تغذیه و استرس از نظر پرستارانN25	سئوال در مورد نوشابه رژیمیP315
			مهم بودن تغذیهN11	تاکید بیماران روی میوهN113
			توصیه کردن تغذیه کمکیN142	مهم بودن میوه برای بیمارانN114
			شک داشتن بیماران در مورد تغذیه کمکیN143	ندانستن وجود چربی در لبنیاتN115
			تغییردادن و اصلاح رژیم غذاییD12	اولویت داشتن تغیه از نظر بیمارانN18
			بومی بودن مطالب مثل غذاهاD226	اینکه چه چیزی بخورندN19
			استفاده از واحدهای غذاییD13	اینکه چقدر بخورندN119
			مهم بودن رژیم غذاییD120	اینکه چه چیزهایی نخورندN111
			مهم بودن تغذیهD23	اینکه چه چیزی برای دیابت خوب استN112
			جالب بودن تغذیهP331	مشکل لاغری و چاقی بیمارانP68

			مهم بودن تشویق غذاییP211<br>پیشنهاد دادن رژیم دیابتیP210<br>تاثیر داشتن نوشابه های رژیمیP320<br>جالب بودن تغذیه و دیابتP626	جالب بودن تغذیه و دیابتP626<br>خواستن لیست غذا برای دیابتیهاP23<br>معرفی کردن محصولات تبلیغی خوراکی دیابتی هاP618<br>وجود داشتن فروشگاه آنلاین محصولات خوراکی P619
	Self-Care illness requirements	Identifying and control of diabetes complications<br><br>شناخت و کنترل عوارض دیابت	مهم بودن پای دیابتیN12<br>مهم بودن عوارض دیابتN13<br>علل ضعف دیابتی هاP325<br>دانستن عوارض قند بالاP323<br>تاثیر روی مسایل جنسیP232<br>مشکل تعریق بیمارانP513<br>مشکل سردرد بیمارانP515<br>مراقبت از دهان و دندانP615<br>درمان زخم های دیابتیP616<br>لزوم دانستن عوارض کلیوی دیابتP311<br>لزوم دانستن عوارض چشمی دیابتP312<br>لزوم دانستن عوارض دهانی دیابتP313<br>قرار دادن مراقبت دندانیD231<br>قرار دادن مشکلات جنسیD232	عوارض کلیه و پای دیابتیp16<br>مشکلات جنسی در خانم های دیابتیp17<br>اضافه شدن عوارض قلبیp18<br>اضافه شدن عوارض کلیویp19<br>اضافه شدن عوارض چشمیp110<br>اضافه شدن بیماریهای دهانp114<br>مهم بودن زخم های دیابتیP25<br>خشکی و خارش پاP218<br>عوارض قند خون پایینP230<br>عوارض قند خون بالاP231<br>سئوال کردن در مورد زخم و پای دیابتیN27<br>ندانستن اینکه پای دیابتی از عوارض استN15<br>آگاهی نداشتن از اینکه سکته قلبی جزء عوارض است N16<br>رعایت نکردن بیماران باعث بروز عوارض شدنN132
	Self-Care illness requirements	Understanding and managing stress and its causes<br><br>شناخت و کنترل استرس و عوامل آن	زیادشدن استرس در کلینیکP412<br>ترس از چک کردن قند خونP324<br>مهم بودن گرفتاریهای زندگیP28<br>هشدار دادنP519<br>کم خواب بودنP416	آموزش تکنیکهای کاهش استرس و ریلکسیشنN240<br>دانستن نحوه کنترل کردن استرسN26<br>مهم بودن اضطرابD21<br>اضافه شدن استرسN154<br>نداشتن تفریحP228

			توانایی کنترل استرسP217<br>مهم بودن خوابP26<br>بالا رفتن فشار با آمدن به کلینیکP413<br>راحت بودن اعصابP215<br>داشتن استرس زیادP411	نداشتن آرامش در مردمP227<br>استرس داشتنP327<br>نگران شدن از قند بالاP329<br>مهم بودن اعصاب P214<br>مشکل بی حوصلگی بیمارانP514<br>ترس از درمان قند بالاP617
	Satisfaction	فعالیت فیزیکی	تاثیرداشتن فعالیتهای بدنی در کنترل دیابتD127<br>مهم بودن ورزش P213	مهم بودن تحرکD22<br>دانستن میزان ورزش و پیاده رویP415<br>جذاب بودن ورش و دیابتP625
	Satisfaction	Medical information	اطلاع نداشتن در مورد داروهاN29<br>چگونه مصرف کردن داروهاN210<br>پایین بودن سطح آگاهیN14<br>نیاز داشتن بیماران به آموزشN17<br>نیاز فرد به آگاهیN116<br>نیاز داشتن بیماران دیابتی به آموزشN117<br>مهم بودن آموزشN119<br>لزوم اطلاع رسانی به بیمارانN135<br>پرسش کردن بیماران در کلاسهاN148<br>دوست داشتن پرسش پاسخ در بیماران مسنN149<br>استفاده از سایت برای پیشگیری از دیابتN146<br>خوب بودن پمفلت های آموزشیN138<br>درخوست کردن پمفلت توسط بیمارانN139<br>پیام در مورد دارو درمانیD113<br>جمع بندی شدن مطالب مورد نیاز بیمارانD117<br>محاسبه کالری مورد نیاز هر بیمارD119	مناسب بودن دیابت و حاملگیP219<br>مهم بودن دیابت و حاملگیP229<br>مهم بودن فشار وچربی خونP225<br>دانستن مفهوم HbA1c P226<br>ندانستنHBA1c P326<br>علاقه مند بودن به بالا رفتن اطلاعات داروییP45<br>علاقه مند بودن به دانستن عوارض داروهاP46<br>دانستن بیشتر در مورد تغذیهP47<br>دانستن میزان قند مطلوبP48<br>داروهای جدیدP517<br>درمانهای جدیدP518<br>دانستن میزان قند غذاهاP322<br>قرار دادن داروهای سنتیP66<br>نیاز به مطالعه بیشترP310<br>علاقمند به اطلاعات پزشکی و داروییP429<br>بررسی برگه آزمایشاتP428

			تغییر دادن سبک زندگیD11<br>آموزش روشها به بیمارانD24	چگونگی روزه داریD121<br>نداشتن وقت برای دکتر رفتنP316
**Access to information**<br>**دسترسی به اطلاعات**		**Patient engagement**<br>**تعامل بیمار**	دادن پیامک برای معرفی سایتP628<br>پاسخگو بودن پزشک بیمارP314<br>مهم بودن ارتباط با پزشکP319<br>لزوم وجود لینک ارتباط با پزشکP510<br>مهم بودن طرز رفتار پزشکP336<br>مناسب بودن پیشرفت سلامتیP221<br>مناسب بودن مرا کمک کنیدP224<br>لزوم به روز بودن مطالبP516<br>تغییر دادن رفتارهای قبل P22<br>گین داشتن برای بیمارانD224<br>گذاشتن گین یا جایزهD28<br>وجود گزینه اخبارD216	اطلاع رسانی در مورد سایت با کمک کلینیکN145<br>موثر بودن تحصیلات بیمارانN147<br>خوب بودن ارسال ایمیلN150<br>بهتر بودن پیامکN151<br>سن بالا داشتن بیماران دیابتیN140<br>کمک کردن فرزندان بیمارانN141<br>ترجیح داردن گفتگوی آنلاینN229<br>خوب بودن گفتگوی بیمارانN234<br>خوب بودن یادآوریN237<br>قرار دادن نظر سنجی بیمارانN218<br>مشغله کاری بیمارانN227<br>کلی گویی نباشدD215
	**Implementation**<br>**پیاده سازی**	**Access to Internet**	مناسب بودن اینترنت برای آموزشN127<br>وجود بیمارانی که دراینترنت مطالبی خوانده اندN128<br>استفاده کم بیماران از اینترنتN211<br>کمتر استفاده کردن بیماران مسن از اینترنتN212<br>دسترسی کم بیماران به اینترنتN213<br>استفاده از سایت در صورت وجود سایت کامل و جامعN214<br>استفاده از اینترنت در بعضی جاهاN215<br>خوب بودن دسترسی بیماران به اینترنتN223<br>دسترسی کم بیماران به اینترنت N224	مشکل نبودن استفاده کردن از سایت p11<br>امکان استفاده با گوشی موبایلp13<br>اضافه شدن مشکل خواب بیمارانp14<br>دانستن کمک به پیشگیری میکندp111<br>مشکل بودن استفاده از اینترنتP61<br>در دسترس نبودن اینترنت در همه جاP627<br>مهم بودن اطلاع رسانیP335<br>مطالعه کردن بیشترP337<br>استفاده کردن از اینترنت P21

			سن بالا بودن بیمارانN225<br>قرار دادن اینترنت در کلینیکN241<br>استفاده کمتر بیماران از اینترنت برای آموزشN226<br>تالار گفتگو چون باید تایپ کنند علاقه ندارندD219<br>مشکل بودن دسترسی به اینترنت در ایرانD132<br>جاذبه نداشتن اینترنتD27	علاقمند به خواندن کتابP222<br>استفاده از اینترنت برای یافتن جواب سئوالاتP41<br>وقت نگذاشتن بیماران برای آموزشD29<br>در دسترس نبودن همیشه اینترنتD18<br>پایین بودن سرعت اینترنتD17<br>عدم امکان نمایش فیلم در سایت هاD19
User interface	user-friendly	Structural Factors<br>عوامل ساختاری	نحوه استفاده کردن از محاسبه گرهاP69<br>مناسب بودن تالار گفتگوP610<br>مناسب بودن وجود پرسش و پاسخ آنلاینP611<br>مناسب بودن اخبار دیابتیP612<br>وجود پرسش و پاسخ آنلاینP613<br>دوست داشتن پرسش و پاسخP425<br>خوب بودن لینکهاP426	خوب بودن متون آموزشیN230<br>خوب بودن وجود وب سایتN231<br>خوب بودن وجود مطالب آموزشیN232<br>خوب بودن وجود فیلم آموزشیN233<br>خوب بودن ارسال پیامکN235<br>طبقه بندی داشتن مطالبN222<br>خوب بودن پیشرفت سلامتیP427<br>تو در تو بودن لینکهاP623
	user-friendly	Customized<br>سفارشی	لزوم وجود اولویت بندی مطالب در سایتN129<br>لزوم وجود توصیه های لازم در سایت برای بیمارانN130<br>امکان دسترسی افراد در معرض خطرN131<br>امکان دسترسی عموم به سایتN133<br>ارسال مطالب جدید در حجم کم به بیمارانN236<br>ساده و روان بودن مطالب آموزشیD136<br>سئوال کردن بیماران از مطالب موجود در اینترنتD128<br>اطلاع نداشتن از مطالعه بیماران از مطالب آموزشی<br>D129	استفاده از روش حل مشکلD225<br>استفاده از سایت در کلینیکD227<br>قرار ندادن موارد خطرناکD228<br>مطالب فعلی پسیو میباشندD14<br>لزوم فعال بودن آموزش دهندهD115<br>یکپارچه بودن مطالب در سایتD116<br>کاربردی بودن مطالبD214<br>راضی بودن بیماران از پیام های آموزشیD130<br>راضی بودن بیماران از یادآوریهاD131
	user-friendly	Options features	مناسبت داشتن مطالب و پیام هاD223<br>وجود امکان پرسش و پاسخD122	ارسال اطلاعات با ایمیل یا پیامکD15<br>مناسب بودن یادآوریD16

			زیاد بودن لینک خوب استD123<br>وجود لینکهای زیادD213<br>وجود دسترسی به مطالب در کلینیکD118<br>مهم بودن اطلاع رسانیD221<br>مهم بودن یادآوریD222<br>مهم بودن نظر بیمارانD230<br>مهم بودن طراحی سایتD212<br>خوب بودن پرسش و پاسخ بیمارانD133<br>وجود داشتن فرمولی برای تعیین کالری مصرفیD139	امکان پرسیدن سئوال بیمارانD110<br>پیام در مورد پیشگیریD112<br>مناسب بودن هفته ای یک تا دو پیامD114<br>مناسب بودن فیلم های آموزشیD134<br>مناسب بودن اصلاید و پاورپوینتD135<br>مشخص شدن دفعات بازدید صفحاتD235<br>مهم بودن امکان پرسش و پاسخD137<br>مناسب بودن فیلم های آموزشیD234<br>دانستن در مورد کفش مناسب برای دیابتی هاP223
	Easy ساده	**Mobile and applications**	مناسب بودن نرم افزارD138<br>نرم افزار ساده مناسب استD111<br>مناسب بودن تلگرام D233<br>داشتن نرم افزار در گوشیBMI P36<br>خوب بودن اپلیکشن های موبایلD211	همراه بودن موبایلP62<br>مناسب بودن آپلیکیشنP64<br>بهتر بودن روی گوشی موبایلP65<br>مناسب بودن روی موبایلP622<br>مفید بودن اپلیکیشنهای موبایلN242
	Easy ساده	**Educational film**	مناسب بودن فیلم هایی که امیدواری میدهندP421<br>دوست داشتن فیلم ها خوب P420<br>مناسب بودن فیلم های آموزشیD220<br>تاثیر بد فیلم های اموزشی ترسانندهP418	دیدن فیلم های اموزشی دیابت در اینترنتP417<br>فیلم ها باید امیدواری بدهندP419<br>مناسب بودن انیمیشن برای بچه هاD26<br>مناسب بودن انیمیشنD217
	Easy ساده	**Entertainment**	کار بردی بودنP38<br>کامل بودن سایتP39<br>علاقمند شدن به مطالعهP32<br>مناسب بودنم مطالبP33<br>پیچیده نبودن شکلهاP34	جالب بودن ابزارP332<br>جالب بودن پیشرفت سلامتیP333<br>کامل بودن سایتP614<br>کامل و مفید بودن سایتP624<br>واضح بودن سایتP31

			خوب بودن تصاویرP410<br>خوانا و خوب بودن خطوطp12<br>خوانا بودن خطوطp15	علاقه مند شدن به خواندن مطالب سایتP334<br>بودن چیزهایی که میخواستهP37<br>اطلاع نداشتن از فرمول BMI P35
	Efficient<br>کارآمد	Implementation	خوب بودن سایت گابریکN124<br>معتبر بودن سایت گابریکN125<br>کامل نبودن سایت گابریکN126<br>توصیه کردن گابریک به بیمارانN136<br>آموزشی بودن سایت های فروش انسولینN137<br>وجود داشتن مطالب آموزشی در سایت های دیگر N216	مناسب بودن اینترنتD25<br>مناسب بودن ایفای نقشD218<br>مناسب بودن شبکه های مجازیD210<br>مناسب بودن فلوچارتD229
	Efficient<br>کارآمد	Visualization	عنوان گذاشتن برای دوره های آموزشیP56<br>استفاده کردن از نمادها روی ایکونهاP57<br>بزرگتر شدن فونتهاP58<br>توضیح دادن ابزارهاP59<br>فشرده بودن عکسهاP55	زیاد بودن عکسهاP51<br>نوشتن زیرنویس برای عکسهاP52<br>نوشتن زیرعنوانهاP53<br>توضیح دادن زیر عکسهاP54
	Efficient<br>کارآمد	Family Support	دادن اطلاعات به سایر افراد فامیل<br>موثر بودن خانوادهP620<br>ارسال پیامک به خانواده بیمارانP621	کمک کردن فرزند برای استفاده از اینترنتP44<br>استفاده کردن فرزند از اینترنتP42<br>کمک همسر در غذاهاP328
		Peer support	قراردادن نظرات بیمارانP511<br>قراردادن سخن شما یا تجارب شماP512<br>گرفتن اطلاعات از دیگرانP220	خوب بودن تبادل تجاربP424<br>یادگیری از دیگر بیمارانP216<br>راهنمایی دادن به دیگرانP423
	Efficient<br>کارآمد	Accuracy<br>دقت	قرار دادن مطالب با توجه به نیاز بیمارانN219<br>به روز بودن مطالبN220<br>معتبر بودن مطالبN221<br>طبقه بندی بودن مطالبN217<br>استقبال بیماران از سایت جامع و کاملN228	اشتباه بودن بعضی از منابع آموزشی در اینترنتN120<br>منابع آموزشی باید موثق باشندN121<br>اشتباه بودن بعضی از منابع آموزشی در اینترنتN122<br>سردرگم شدن بیماران با اطلاعات در اینترنتN123<br>نداشتن آگاهی بیماران N134

SubCategories	Categories	Themes
Suitable nutrition in diabetes تغذیه صحیح در دیابت	Self-Care Requirements الزامات خودمراقبتی بیماری	Improvement Self-care
Physical فعالیت فیزیکی activity		
Identifying and control of diabetes complications شناخت و کنترل عوارض دیابت	Management	
Understanding and managing stress and its causes شناخت و کنترل استرس و عوامل آن		
Medical information	Satisfaction رضایت	Access to information
Educational film		
Patient engagement تعامل بیمار	Implementation پیاده سازی	
Access to Internet		
Structural Factors عوامل ساختاری	user-friendlyلذت بخش	User interface رابط با کاربر بودن
Customization سفارشی نمودن		
Options features		
Using mobile and applications	Easyساده	
Entertainment		
Implementation	Efficientکارآمد	
Visualization		
Family Support		
Peer support		
Accuracy		

طبقه بندی کدهای مصاحبه ها ی مرحله سوم

	Sub Categories	Categories	codes
	رضایت مندی از سایت<br>**Satisfaction from the website**	خوب و مفید بودن سایت<br><br><br>کامل بودن سایت	D1خوب بودن کلیت سایت<br>N1کامل بودن و خوب بودن<br>P2خوب بودن مطالب<br>P2مفید بودن سایت<br>P4خوب و عالی بودن<br>P6خوب بودن سایت<br>P19خوب بودن سایت<br>P6مفید بودن سایت<br>P7عالی بودن سایت<br>P9خوب بودن سایت<br>P10خیلی خوب بودن سایت<br>P11جالب بودن سایت<br>P16خیلی خوب و عالی بودن سایت<br><br>P15خیلی کامل بودن سایت<br>P15خیلی کامل بودن سایت<br>P17کامل بودن سایت<br>P18کامل بودن سایت<br>P9تکمیل بودن سایت<br>P6کامل بودن سایت<br>P8کامل بودن سایت<br>P10نرسیدن چیز جدید به ذهن<br>P17کاملتر شدن سایت نسبت به قبل<br>P9نداشتن نظر جدید<br>P10مناسب بودن طراحی سایت
	تمایل به استفاده از سایت<br>**Desire to use the website**	ارایه نظرات پس از استفاده	P1نظر دادن بیشتر بعد از استفاده<br>P8استفاده کردن و بعد نظر دادن<br>P7نظر دادن بعد از استفاده کردن

P13نظر دادن بعد از استفاده کردن<br>P18پیشنهاد دادن بعد از شروع استفاده<br>P7با حوصله خواندن و بعد نظر دادن<br><br>D1اضافه کردن جزییات در آینده<br>D1استفاده کردن و حل مشکلات<br>P9حتما استفاده کردن<br>P9تمایل داشتن به استفاده هرچه زودتر<br>P10مشخص شدن مشکلات در آینده<br>P16عجله داشتن برای استفاده از سایت<br>P11عجله داشتن برای استفاده<br>P9تشکر کردن از راه اندازی سایت	رغبت کردن استفاده از وب سایت		
N1خوب بودن گفتگور انلاین<br>P2مناسب بودن اینکه اینترنتی است<br>P13خوب بودن در دسترس بودن<br>P10راحت و در دسترس بودن<br>P4دسترسی داشتن آسان<br>P18مفید بودن از نظر هزینه مالی<br><br>P4خوب بودن آموزش<br>P14خوب بودن دوره های آموزشی<br>D1مناسب بودن فیلم های آموزشی<br>P6مفید و جالب بودن دیابت و ارگانها<br>P9خوب بودن دیابت و ارگانها<br>P12خوب بودن دیابت و کلیه<br>P11مناسب بودن برای پیشگیری<br>P19امکان راهنمایی کردن بیمار<br>P12خوب بودن کمکم کنید<br>P11خوب بودن تغذیه و دیابت<br>P13کاربردی بودن تغذیه و دیابت<br>P17خوب بودن اطلاعات	دسترسی آسان<br><br>آموزش مناسب	کار آمد بودن<br>Efficient	

P19مناسب بودن پیشرفت سلامتی<br>P19امکان کنترل کردن بیمار با سایت<br>P18خوب بودن جواب به سئوالات<br>P8جالب بودن رسم نمودار<br>P12عالی بودن نمودارها<br>P15خیلی خوب بودن پیشرفت سلامتی<br>P12خوب بودن پیشرفت سلامتی<br>P14خیلی خوب بودن نمودارها در پیشرفت سلامتی<br>P4تکمیل بودن آزمایشات	پسخوراند مناسب		
P9مناسب بودن ارتباط با پزشک و پرستار<br>P8خیلی خوب بودن ارتباط با پزشک<br>P13مناسب بودن ارتباط با پزشک<br>P10عالی بودن ارتباط با پزشک<br>P19مفید بودن سایت برای پزشکان<br>P14خیلی خوب بودن ارتباط با پزشک<br>P18خوب بودن ارتباط با پزشک<br>P18راحت بودن ارتباط با پزشک<br>D2دسترسی داشتن آسان به سایت<br>P18امکان نوبت دهی با سایت<br>P13مهم بودن حضور مدریتور در گروه	ارتباط مناسب		
P14خیلی خوب بودن محاسبه کالری<br>P11خوب بودن محاسبه کالری<br>N1خوب بودن جدول کالری<br>P4جالب بودن محاسبه گرها<br>P8خوب بودن محاسبه کالری<br>P13خوب بودن محاسبه کالری<br>P10کاربردی بودن محاسبات<br>P10خوب بودن منوهای سایت	راهنمای منلسب		

	Add structural factors<br><br>اضافه کردن عوامل ساختاری	سلسله مراتب بصری<br>Visual Hierarchy	D1اصلاح کردن آزمایش<br>D1اضافه کردن لینکها<br>P4ریز بودن خط<br>P4اضافه کردن آیکون اطلاعات جدید<br>P4عوض کردن عکس تاریخ تقریبی زایمان<br>P4لزوم گویا بودن تصاویر<br>P4نشان دادن در قالب شکل و نمودار<br>D2قراردادن راهنمای مصرف دارو<br>D2قراردادن راهنمای مصرف دارو
		سادگی<br>Simplicity	P4ساده شدن جداول<br>P10بیشتر کردن قسمت ویژوال در سایت<br>P10امکان خواندن متن برای بیماران<br>P10تهیه کردن ویدوهای آموزشی
		Accessibility<br>دسترسی	D1گذاشتن مطالب آموزشی برای پزشکان<br>D1امکان استفاده کرده بصورت آفلاین<br>D1گذاشتن هزینه آزمایشات و داروها<br>N1دسترسی داشتن پرستار به ازمایشات بیماران<br>D2قادر بودن پزشک به دسترسی به خلاصه پرونده بیمار<br>D2نشان دادن خلاصه پرونده بیمار به پزشک<br>D1گذاشتن داروهای دیابتی برای اطلاع<br>D2دسترسی داشتن بیماربه اطلاعات دارویی<br>P1گذاشتن برنامه غذایی هفتگی<br>D2ثبت کردن داروهای بیمار<br>D2قابلیت داشتن سیستم برای تشخیص تداخلات دارویی<br>P18امکان ایمیل زدن به دکتر<br>D1قابلیت ایمیل کردن به بیمار

		User-Centricity<br>کاربر مداری	P4نحوه کنترل کردن مشکل عصبی<br>P7نگران بودن درخصوص کلیه<br>P15علاقه به دانستن تاثیر استرس روی دیابت<br>P17گذاشتن مطالب در خصوص کنترل استرس<br>N1مهم بودن افت قندخون.استرس.پا<br>P7دوست داشتن اطلاعات در مورد بیماری<br>D2قرار دادن سایر بیماریهایی که بیمار دارد<br>P2اطلاعات خواستن در مورد دیابت و حاملگی
		Credibility<br>اعتبار داشتن	D1گذاشتن لینکهای مفید برای پزشکان<br>D1امکان پخش موسقی همزمان با مطالعه<br>D2ورود کردن به سایت با کد ملی<br>D2دسته بندی کردن داروهای مصرفی بیمار<br>D2ثبت کردن ارجاعات و پاراکلینیک<br>D2آلارم دادن مراجعه بعدی به بیمار<br>N1اصلاح کردن نام آزمایشات<br>N1حق انتخاب داشتن دوره های آموزشی<br>P14قرار دادن گیاهان دارویی و دیابت<br>P15گذاشتن مطلب برای افراد در معرض<br>P18توضیح دادن در مورد آزمایشات<br>P18امکان مشورت و پیام گذاشتن به دکتر
	تاکید بر اصلاح<br>**Emphasizing to modify**	ایجاد جذابیت	D1مهم بودن جذابیت<br>P10مناسب بودن برای جوانترها<br>P10سخت جذب شدن افراد مسن به سایت<br>P15مفید بودن برای خانم های خانه دار
		پاسخگو بودن به بیماران	P15اهمیت سریع جواب دادن پزشکان و پرستاران<br>D1مهم بودن سئوال و جواب<br>P16ساده بودن مطالب مهم است

P16مهم بودن سریع جواب دادن پزشک<br>P15مهم بودن آنلاینی پزشکان و پرستاران<br>P18مهم بودن پاسخ به سئوالات توسط دکتر<br><br>P10مهم بودن آپدیت شدن سریع<br>P16پیشنهاد بودن چند پزشک در سایت<br>P18فعال بودن ارتباط سایت با بیماران<br>P16اطمینان داشتن از آنلاین بودن	پاسخگویی سریع		
D1جذاب کردن مطالب برای بیماران<br>D1آلارم دادن برای مدت استفاده از سایت<br>N1گذاشتن راهنمای استفاده<br>P3خود بخود وارد شدن اطلاعات درسایت<br>P4ساده شدن دسترسی<br>P4قابل فهم بودن برای همه<br>P4امکان استفاده همگان<br>P11امکان خوانده شدن مطالب<br>P16استفاده نکردن از اصطلاحات پزشکی<br>P18گذاشتن توضیحات برای آزمایشات<br>P18توضیح دادن شمارش کربوهیدرات<br>P18محاسبه کردن کالری توسط سایت<br>P19 واردکردن آزمایشات توسط سایت<br>P10وقت گرفتن از طریق سایت	آسان برای استفاده<br><br>کاربردی بودن	لذت بخش ساختن<br>Making user friendly	

طبقه بندی نهایی حاصل شده ار مصاحبه های مرحله دوم

Themes	Categories	Sub Categories
آماده برای استفاده<br>waiting to use	رضایت مندی از سایت<br>Satisfaction from the website	خوب و مفید بودن سایت
		کامل بودن سایت
	تمایل به استفاده از سایت<br>Desire to use the website	ارایه نظرات پس از استفاده
		رغبت کردن استفاده از وب سایت
	کار آمد بودن<br>Efficient	دسترسی آسان
		آموزش مناسب
		پسخوراند مناسب
		ارتباط مناسب
		راهنمای منلسب
	لذت بخش ساختن<br>Making user friendly	آسان برای استفاده
		کاربردی بودن
انجام تغییرات<br>Making changes	Add structural factors<br>اضافه کردن عوامل ساختاری	سللسله مراتب بصری<br>Visual Hierarchy
		سادگی<br>Simplicity
		Accessibility<br>دسترسی
		User-Centricity<br>کاربر مداری
		Credibility<br>اعتبار داشتن
	تاکید بر اصلاح<br>Emphasizing to modify	ایجاد جذابیت
		پاسخگو بودن به بیماران
		پاسخگویی سریع
